# Pharmacogenetics-Guided De-Escalation of Dual Antiplatelet Therapy Based on CYP2C19 Testing in Patients After Acute Coronary Syndrome: Clinical Outcomes and Cost–Consequence Analysis

**DOI:** 10.3390/jcm15187261

**Published:** 2026-09-18

**Authors:** Firuz Kh. Turaev, Sherzod P. Abdullaev, Kirill I. Matrenin, Furkatjon S. Gafurov, Svetlana N. Tuchkova, David A. Gabrielyan, Karin B. Mirzaev, Dmitry A. Kaprin, Dmitry A. Sychev

**Affiliations:** 1 Egoryevsk Hospital of the Moscow Region, Egoryevsk 140301, Russia; 2 Petrovsky National Research Center of Surgery, Moscow 119435, Russia; 3 Center for Healthcare Quality Assessment and Control, Ministry of Health of the Russian Federation, Moscow 109028, Russia; 4 Russian Medical Academy of Continuous Professional Education, Moscow 125993, Russia

**Keywords:** CYP2C19, clopidogrel, ticagrelor, antiplatelet pharmacotherapy, personalized medicine, pharmacogenetics, genetics

## Abstract

**Background/Objectives**: De-escalation of dual antiplatelet therapy (DAPT) from ticagrelor to clopidogrel after percutaneous coronary intervention (PCI) for acute coronary syndrome (ACS) reduces bleeding risk; however, in carriers of loss-of-function CYP2C19 alleles, it may carry a risk of insufficient platelet inhibition. This study compared the safety and efficacy of CYP2C19 genotype-guided DAPT de-escalation with standard clinical practice in patients after ACS and PCI, and evaluated the cost implications of this approach. **Methods**: We conducted a prospective, randomized, open-label, parallel group trial (*n* = 75 genotype-guided group; *n* = 75 control group) with 12 months of follow-up. In the genotype-guided group, the de-escalation decision was based on the CYP2C19 genotype; in the control group, it followed standard practice at the treating physician’s discretion. The primary safety endpoint was the cumulative incidence of clinically relevant bleeding (BARC ≥2); the secondary efficacy endpoint was a composite of ischemic events. The economic evaluation was performed as a cost–consequence analysis (CCA), reporting drug and testing costs alongside the clinical outcomes described above, including a break-even threshold and one-way sensitivity analysis. **Results**: In the intention-to-treat population (*n* = 150), the 12-month cumulative incidence of BARC ≥ 2 bleeding was 10.7% in the genotype-guided group versus 12.0% in the control group (HR 0.88; 95% CI 0.34–2.28; *p* = 0.79). The cumulative incidence of the ischemic composite was 13.3% versus 14.7% (HR 0.91; 95% CI 0.38–2.13; *p* = 0.82), and neither difference was statistically significant. A per-protocol sensitivity analysis excluding eight patients who required non-protocol anticoagulant therapy yielded similar results for bleeding (9.7% vs. 11.4%; HR 0.84) and a numerically larger, though still non-significant, effect for the ischemic composite (11.1% vs. 15.7%; HR 0.69). Use of the genotype-guided strategy was associated with savings in drug and pharmacogenetic testing costs of RUB 1,036,340.21 for the cohort (RUB 15,278.16 per patient), a saving that remained positive across the full range of observed drug prices; the drug-and-testing-cost break-even price for testing was RUB 19,748.16 per patient. **Conclusions**: No statistically significant differences in bleeding or ischemic events were detected between genotype-guided de-escalation of DAPT based on CYP2C19 testing and standard practice in this trial, and the genotype-guided strategy was associated with a reduction in drug and testing costs. Confirmation of the clinical benefit of this approach requires further studies in larger cohorts.

## 1. Introduction

Acute coronary syndrome (ACS) remains one of the leading causes of death worldwide. According to the Global Burden of Disease Study, cardiovascular disease accounted for 18.6 million deaths in 2019, of which 9.14 million were attributable to ischemic heart disease (IHD), including ACS [1]. In Europe, approximately 4 million cardiovascular deaths are recorded annually, with ACS accounting for up to 20% of these [2]. In the United States, the American Heart Association (AHA) reports approximately 805,000 myocardial infarctions annually, 15% of which are fatal within the first year [3]. In Russia, despite a decline in cardiovascular mortality over the past decade, ACS continues to hold a leading position: according to Rosstat data for 2022, mortality from IHD, including ACS, accounted for 584,000 deaths, corresponding to 45% of all cardiovascular deaths [4].

Percutaneous coronary intervention (PCI) is the primary revascularization method for patients with ACS, substantially improving prognosis by restoring coronary blood flow [5]. However, the long-term success of the procedure depends directly on the effectiveness of secondary prevention, in which dual antiplatelet therapy (DAPT) plays a central role. According to Russian and international guidelines, the combination of acetylsalicylic acid and a P2Y12 receptor inhibitor such as clopidogrel minimizes the risk of thrombotic complications after PCI [6,7]. Despite its widespread use, clopidogrel shows marked inter-individual variability in antiplatelet effect, related to genetic polymorphism of cytochrome CYP2C19, the enzyme responsible for converting the prodrug into its active metabolite. Carriers of loss-of-function CYP2C19 alleles (e.g., *2 or *3) have reduced clopidogrel metabolism, resulting in insufficient platelet inhibition due to decreased formation of the active metabolite; this, in turn, increases the risk of recurrent ischemic events [8,9].

According to the large randomized controlled trial (RCT) PLATO, ticagrelor is superior to clopidogrel in reducing ischemic events in patients with ACS [10]. However, this benefit comes at the cost of a higher risk of hemorrhagic complications, including non-CABG-related major bleeding and spontaneous bleeding [11]. Even when non-fatal, these bleeding events place a substantial financial burden on the healthcare system through hospitalizations, transfusions, diagnostic procedures, and unscheduled consultations.

One approach to reducing bleeding risk during ticagrelor therapy is de-escalation: switching to a P2Y12 inhibitor with lower inhibitory activity after a defined interval following discharge [12,13]. This strategy can potentially reduce bleeding rates, but introduces a risk of ischemic complications related to genetically determined variability in clopidogrel metabolism.

In clinical practice, the decision to de-escalate antiplatelet therapy is most often made on clinical grounds and factors, without genotype data. An alternative is a pharmacogenetically guided strategy, in which the decision to switch to clopidogrel is made after testing for CYP2C19 alleles affecting drug metabolism. Despite recommendations from professional pharmacogenetics societies such as CPIC (Clinical Pharmacogenetics Implementation Consortium) and DPWG (Dutch Pharmacogenetics Working Group) to adjust antiplatelet therapy after ACS/PCI in carriers of loss-of-function CYP2C19 alleles [14,15], routine genetic testing remains uncommon and is rarely incorporated into clinical standards for ACS management after PCI [16,17,18]. This reflects several factors. On the one hand, the results of large randomized trials remain inconsistent: in the POPular Genetics trial, a genotype-guided strategy showed no benefit in reducing ischemic events [19], whereas TAILOR-PCI showed a trend toward improved outcomes [20]. On the other hand, implementation of testing faces practical barriers, including delays in obtaining results, cost, and limited clinician familiarity with pharmacogenetic algorithms [21]. Current ESC and ACC/AHA guidelines emphasize that the decision to test should be individualized, taking into account resource availability and center experience [16,18], reflecting the need for further research to optimize the approach. An exception is the United Kingdom, where the National Institute for Health and Care Excellence (NICE) launched a CYP2C19 genotyping initiative for patients at risk of vascular events in 2022 and, in 2024, issued formal guidance on CYP2C19 testing to personalize clopidogrel therapy in patients after ischemic stroke or transient ischemic attack [22]. In December 2025, UK CERSI-PGx issued extended recommendations proposing universal CYP2C19 testing before prescribing clopidogrel for any indication [23].

The aim of this study was to comparatively assess the safety and efficacy of CYP2C19 pharmacogenetically guided de-escalation of dual antiplatelet therapy from ticagrelor to clopidogrel, compared with standard clinical practice, in patients after ACS and percutaneous coronary intervention, and to evaluate the cost implications of this approach.

## 2. Materials and Methods

### 2.1. Ethical Considerations

The study was approved by the local ethics committee of the Russian Medical Academy of Continuous Professional Education, Ministry of Health of the Russian Federation (Protocol No. 6, 3 June 2024). The study was conducted in accordance with Russian legislation and applicable international regulatory documents: Declaration of Helsinki of the World Medical Association, 2013; Russian National Standard GOST R 52379-2005.

All patients who took part in the study provided written informed consent. Before consent was obtained, every patient received an explanation of all aspects of the study protocol and its risks and had the opportunity to ask questions.

### 2.2. Study Design

**Figure 1:**
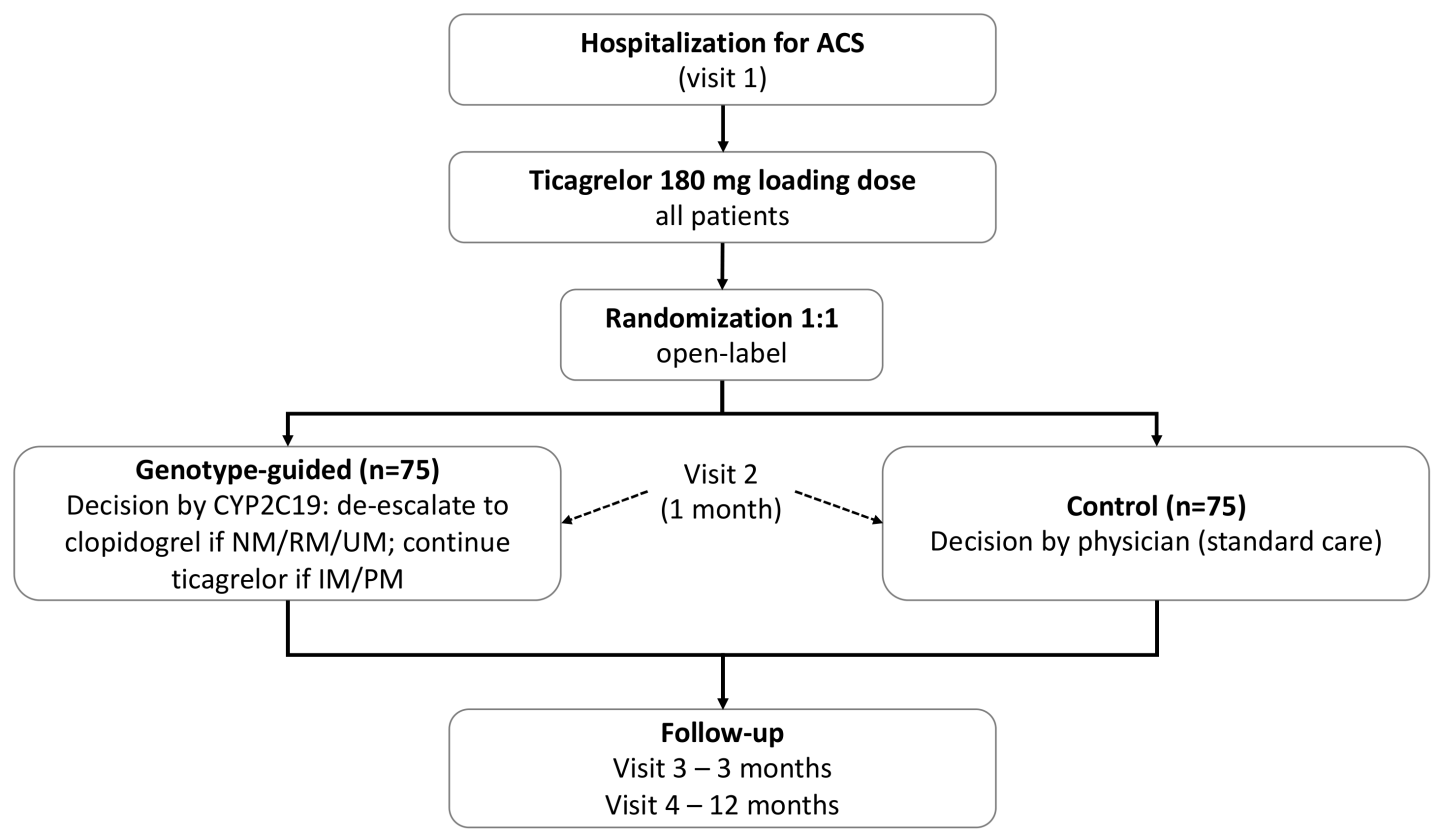
Study design flow chart.

This was a prospective, randomized, open-label, parallel group trial (see Figure 1).

The study enrolled patients hospitalized in the cardiology department of Egoryevsk Hospital, Moscow region; the enrollment period was October 2024 to May 2026.

The enrollment visit (Visit 1) was defined as the time of hospitalization for ACS. All hospitalized patients received a loading dose of ticagrelor, 180 mg, followed by maintenance ticagrelor, 90 mg, twice daily as part of DAPT (in combination with acetylsalicylic acid) during the first month of follow-up, irrespective of randomization group.

The allocation sequence (1 or 2, 1:1 ratio) was generated in advance using the RAND() function in Microsoft Excel by one of the co-authors (Sh.P.A.), a researcher responsible for sample genotyping who was not involved in patient enrollment; each consecutively enrolled patient was assigned to the group corresponding to the next unused value in the sequence. Patients were enrolled by one of three treating physicians at Egoryevsk Hospital, who confirmed eligibility and obtained informed consent without access to the allocation sequence; group assignment was communicated to the enrolling physician by Sh.P.A. only after enrollment, preventing enrolling physicians from anticipating the next allocation in advance. Blinding was not implemented given the open-label nature of the intervention.

Before hospital discharge, venous blood was drawn from all patients, regardless of group, for genotyping of CYP2C19 allelic variants (*2, *3, and *17).

Visit 2 (1 month) was an in-person visit comprising clinical examination, history and symptom review, and the decision on antiplatelet therapy adjustment:

- In the genotype-guided group (*n* = 75), the decision was based on the CYP2C19 genotyping results available by this visit: in the absence of loss-of-function CYP2C19 alleles, patients were de-escalated to clopidogrel, 75 mg, once daily; carriers remained on ticagrelor, 90 mg, twice daily.
- In the control group (*n* = 75), the de-escalation decision was made by the treating physician based on standard clinical criteria (age, bleeding risk, and treatment tolerability), without genotyping results.

Visit 3 (3 months) was a telephone interview assessing symptoms, treatment adherence, and clinical events (hemorrhagic and ischemic complications, rehospitalizations).

Visit 4 (12 months) was a telephone interview equivalent in content to Visit 3, assessing long-term clinical outcomes and completing the follow-up period.

### 2.3. Inclusion/Exclusion (Non-Inclusion) and Discontinuation Criteria

Inclusion criteria:

1. Confirmed diagnosis of acute coronary syndrome (ST-elevation or non-ST-elevation) at the pre-hospital or hospital stage.
2. Percutaneous coronary intervention with stenting of coronary arteries.
3. Administration of a P2Y12 inhibitor loading dose (ticagrelor, 180 mg) pre-hospital, intraoperatively, or within the first day of hospitalization, followed by a maintenance dose (ticagrelor, 90 mg, twice daily) during the first month of follow-up until the de-escalation decision.
4. Concomitant acetylsalicylic acid as part of DAPT.
5. Age ≥ 18 years.
6. Signed informed consent.

Exclusion (Non-Inclusion) criteria:

1. Documented allergic reaction to clopidogrel or ticagrelor.
2. Severe hepatic impairment (Child–Pugh class B or C).
3. Hemorrhagic syndrome.
4. Active clinically significant bleeding at enrollment.
5. History of intracranial hemorrhage (within the preceding 6 months).
6. Active peptic ulcer disease.
7. Pregnancy or breastfeeding.
8. Concomitant use of anticoagulants (warfarin, direct oral anticoagulants) or antiplatelet agents (prasugrel) other than those specified in the protocol.

Discontinuation criteria:

1. Patient withdrawal from the study.
2. Non-adherence to antiplatelet therapy.
3. Switch to a P2Y12 inhibitor not specified in the protocol.
4. Serious adverse events requiring treatment discontinuation.
5. New-onset atrial fibrillation during follow-up requiring anticoagulation (patients were retained in the primary intention-to-treat analysis and excluded only from the per-protocol sensitivity analysis, with the date and reason for withdrawal recorded).
6. Pregnancy occurring during the study.
7. Missing two or more scheduled visits.

### 2.4. Sample Size

The sample size was determined by the practical constraints of recruitment and prospective follow-up at a single center; no formal a priori power calculation was performed for a specific clinical endpoint. A post hoc assessment indicates that the chosen sample size has limited power for relatively rare binary clinical events (bleeding, recurrent ischemic complications); based on data from large international trials [19], detecting reliable differences in these events would require a substantially larger sample. Accordingly, the study was exploratory (pilot) in nature with respect to comparative clinical event rates, while being adequately powered for the pharmacogenetic and cost-related objectives.

### 2.5. Genotyping

From each patient, 5 mL of venous blood was collected on the day of enrollment into single-use sterile EDTA vacuum tubes for subsequent genotyping. Sample collection was performed together with routine blood draws and required no additional venipuncture. Samples were frozen at −20 °C, transported to the laboratory, and subsequently stored at −70 °C.

CYP2C19 allelic variants (*2, rs4244285; *3, rs4986893; *17, rs12248560) were genotyped in all enrolled patients by allele-specific real-time PCR, using commercial kits from Syntol LLC (Moscow, Russia) on a CFX96 Touch™ Real-Time PCR Detection System (Bio-Rad, Hercules, CA, USA).

Diplotype-to-phenotype assignment followed the CPIC 2022 CYP2C19 activity score framework [14]. Normal, rapid, and ultrarapid metabolizers (NM/RM/UM) all retain normal or increased enzymatic conversion of clopidogrel to its active metabolite and were therefore considered eligible for de-escalation to clopidogrel; intermediate and poor metabolizers (IMs/PMs), who carry loss-of-function alleles associated with reduced active metabolite formation, remained on ticagrelor.

### 2.6. Outcomes

The primary safety endpoint was the cumulative incidence of clinically relevant bleeding (BARC ≥ 2, per the Bleeding Academic Research Consortium classification [24]) over 12 months of follow-up. The secondary efficacy endpoint was the cumulative incidence of a composite of ischemic events, comprising cardiovascular death and recurrent ischemic events (unstable angina, acute coronary syndrome, and ischemia-related rehospitalization).

Additional endpoints included: the type distribution of hemorrhagic complications and BARC grade at each visit; the rate and structure of unplanned rehospitalizations; and concordance between the treatment actually received and the CYP2C19 genotype, and its relationship to bleeding and ischemic event rates.

The primary analysis was performed on an intention-to-treat (ITT) basis, including all 150 randomized patients according to their assigned strategy, regardless of subsequent treatment modifications. A pre-specified per-protocol sensitivity analysis excluded patients who initiated anticoagulant therapy for a new indication (atrial fibrillation or left ventricular thrombosis) unrelated to the study de-escalation strategy.

Ischemic events were classified into predefined categories differing in diagnostic certainty: cardiovascular death; recurrent MI with stent thrombosis; angiographically confirmed ACS/unstable angina requiring revascularization; angiographically confirmed ACS/unstable angina not requiring revascularization; and unstable angina diagnosed clinically without repeat angiography. No strokes occurred during follow-up and stroke was not part of the composite definition.

### 2.7. Clinical-Economic Analysis

Because the present trial was not powered to demonstrate clinical equivalence or non-inferiority between the genotype-guided and control strategies, a cost-consequence analysis (CCA) was performed rather than a cost-minimization analysis. This approach reports the costs of each strategy alongside their observed clinical outcomes, without requiring an a priori assumption of comparable efficacy or safety [25].

The analysis was performed from the healthcare system perspective and included direct costs of P2Y12 inhibitor drug therapy and CYP2C19 pharmacogenetic (PGx) testing. The costs of acetylsalicylic acid were not included, as there was no difference in its use between groups. The analysis did not include costs related to hospitalization, repeat revascularization, bleeding management, laboratory monitoring, adverse event treatment, or PGx test implementation and turnaround time.

Drug costs were calculated from prices in the Russian State Register of Maximum Manufacturer Selling Prices for medicines (GRPOTs) [26]—a national regulatory price registry covering drugs on the Russian List of Vital and Essential Medicines—for registered formulations at 90 mg for ticagrelor and 75 mg for clopidogrel, matching the dosing regimens used in the study (ticagrelor, 90 mg, twice daily; clopidogrel, 75 mg, once daily). The price per mg of active substance was calculated as the median across all registered entries in the register for the corresponding INN.

The costs were calculated based on the treatment actually received by each patient (ticagrelor or clopidogrel, depending on whether de-escalation occurred), irrespective of randomization group, over an 11-month follow-up period. This 11-month window spans the period from the de-escalation decision (Visit 2, 1 month) to the end of the 12-month follow-up; the initial month, during which all patients received ticagrelor per protocol regardless of group, was excluded because it does not differ between arms and does not affect the between-group cost difference.

### 2.8. Statistical Analysis

Statistical analysis was performed in R. Normality of distribution was assessed using the Kolmogorov–Smirnov test. Continuous variables were compared using the Mann–Whitney U test (independent groups) and Wilcoxon signed-rank test (paired groups). Categorical variables were compared using Pearson’s chi-square test or Fisher’s exact test. Cumulative incidence of clinical events over follow-up was estimated by the Kaplan–Meier method, with groups compared using the log-rank test. Cox proportional hazards regression was used to estimate hazard ratios, with the proportional hazards assumption assessed via Schoenfeld residuals.

Both the intention-to-treat and per-protocol analyses used identical statistical methods; the results are reported for both populations for each clinical endpoint.

Differences were considered statistically significant at *p* < 0.05.

## 3. Results

### 3.1. Analysis of Clinical, Demographic, and Laboratory Parameters

**Figure 2:**
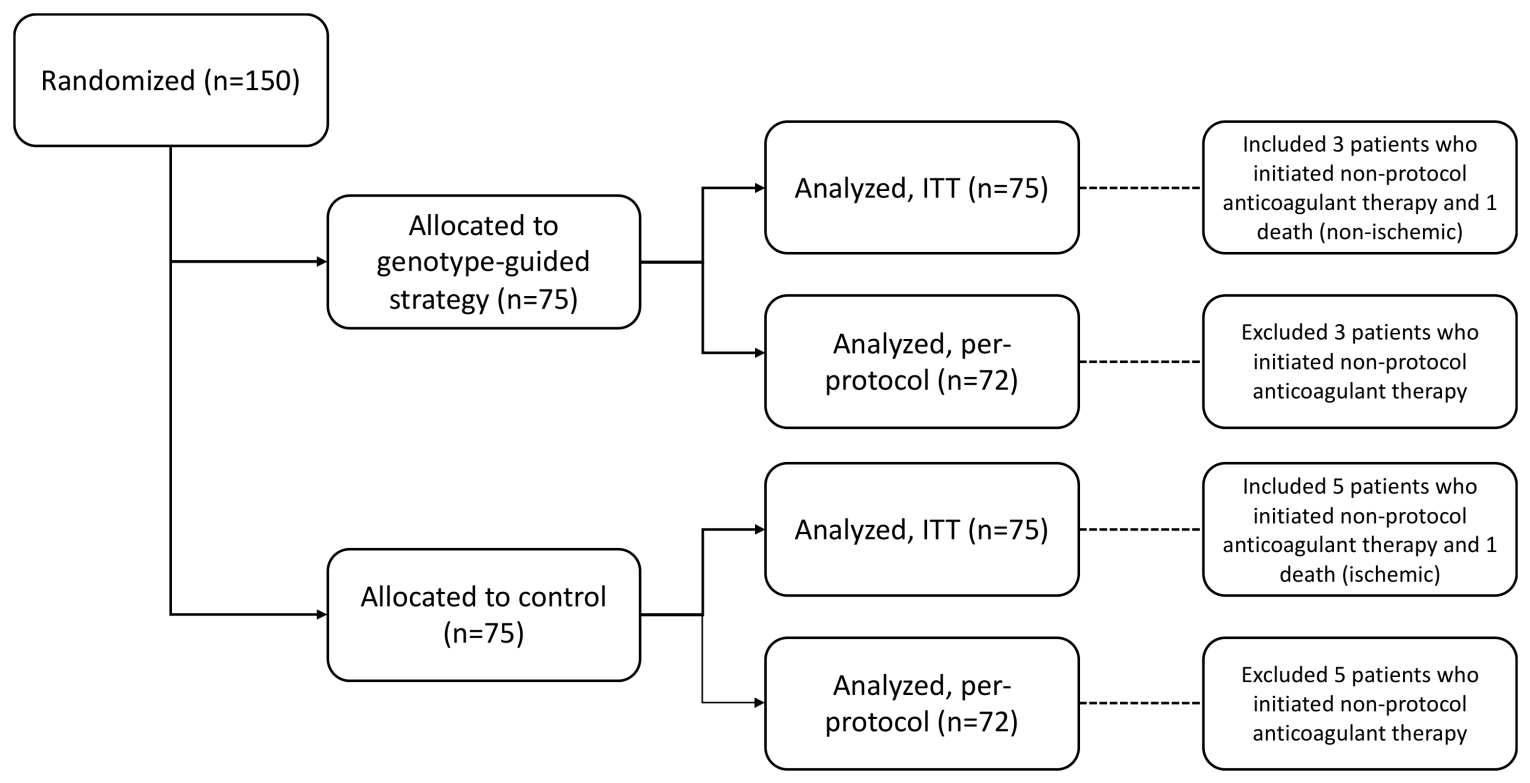
CONSORT flow diagram: randomization, post-randomization treatment modifications, and analysis populations.

The study enrolled 150 patients with acute coronary syndrome who underwent percutaneous coronary intervention with coronary stenting: 75 patients in the genotype-guided group and 75 in the control group (Figure 2).

Median age in the overall cohort was 64.0 years [54.3; 69.0], and men accounted for the majority of the sample (100 patients, 66.7%). ST-elevation ACS was diagnosed in 63 patients (42.0%) and non-ST-elevation ACS in 87 patients (58.0%).

The groups were comparable across most demographic, clinical, angiographic, and laboratory parameters (Table 1): no statistically significant between-group differences were found for age, sex, body mass index, smoking status, comorbidities and treatment history, Killip class at admission, angiographic pattern of coronary involvement, or most laboratory parameters (*p* > 0.05 for all comparisons).

Statistically significant between-group differences were found for three parameters: hemoglobin (140.83 ± 18.14 g/L in the genotype-guided group vs. 147.83 ± 16.28 g/L in the control group, *p* = 0.014), erythrocyte count (4.64 ± 0.57 × 10^12^/L vs. 4.88 ± 0.64 × 10^12^/L, *p* = 0.015), and heart rate at admission (68.0 [65.0;78.0] vs. 72.0 [67.0;82.5] bpm, *p* = 0.048). These differences were not considered likely to materially affect the clinical efficacy or safety of the therapy regimens under comparison.

All patients were genotyped for the three CYP2C19 allelic variants; genotype frequencies are shown in Table 2. The CYP2C19*2 allele was identified in 29 patients: 26 heterozygotes (GA, 17.3%) and three homozygotes (AA, 2.0%), corresponding to a minor allele frequency of 10.7%. The CYP2C19*3 allele was rare, with only one heterozygous carrier (0.7%; minor allele frequency 0.3%). The CYP2C19*17 allele, associated with accelerated metabolism, was identified in 67 patients (44.7%): 56 heterozygotes (37.3%) and 11 homozygotes (7.4%), corresponding to a minor allele frequency of 26.0%.

Genotype distributions for all three variants did not deviate from the Hardy–Weinberg equilibrium (*p* = 0.268 for CYP2C19*2; *p* = 0.715 for CYP2C19*17), which is consistent with the absence of genotyping error.

Based on the identified genotypes, patients were classified into five CYP2C19 metabolizer phenotype categories as per the CPIC recommendations (Table 3). Normal metabolizers (NMs) and rapid metabolizers (RMs) predominated in the overall cohort: 61 (40.7%) and 48 (32.0%), respectively. Intermediate metabolizers (IMs) accounted for 27 patients (18.0%), ultrarapid metabolizers (UMs) for 11 patients (7.3%), and poor metabolizers (PMs) for three patients (2.0%).

The phenotype distribution was comparable and did not differ significantly between groups (Fisher’s exact test, *p* = 0.489): the proportion of patients with an NM/RM/UM phenotype, for whom PGx testing would support de-escalation to clopidogrel, was 81.3% (61/75) in the genotype-guided group and 78.7% (59/75) in the control group, indicating no imbalance between groups.

Concordance between the actual DAPT de-escalation decision and CYP2C19 genotyping results in both study groups is shown in Table 4.

Within 1 month of discharge, eight patients developed atrial fibrillation (*n* = 7) or left ventricular thrombosis (*n* = 1). Because these patients were switched to combined antithrombotic therapy outside the study protocol (rivaroxaban plus clopidogrel or warfarin plus clopidogrel), they were retained in the primary intention-to-treat analysis and excluded only from the per-protocol sensitivity analysis (Section 3.2 and Section 3.3).

In the genotype-guided group, the actual decision matched the genotype-based recommendation in 97.2% of cases; deviations occurred in two patients: one rapid metabolizer (de-escalation erroneously withheld despite no contraindication) and one intermediate metabolizer (de-escalation erroneously performed against the PGx result). In the control group, concordance between the actual decision and the genotype-based recommendation was markedly lower (25.7%): the great majority of discordant cases represented continuation of ticagrelor in patients for whom de-escalation would have been genotype-indicated.

Table 5 reports the treatment actually received at each follow-up visit. The difference in de-escalation rate observed at 1 month persisted throughout follow-up, with substantially more genotype-guided patients receiving clopidogrel and more control patients remaining on ticagrelor at both 3 and 12 months.

### 3.2. DAPT Safety

DAPT safety was assessed based on the rate and structure of hemorrhagic complications by BARC grade over 12 months of follow-up. The primary analysis followed the intention-to-treat principle and included all 150 randomized patients (75 per group). A per-protocol sensitivity analysis additionally excluded eight patients who switched to anticoagulant therapy outside the de-escalation strategy under study, yielding an analysis population of 142 patients (72 genotype-guided group, 70 control group).

Bleeding of any type was recorded in 61 of 142 patients (43.0%) over follow-up: 29 of 72 (40.3%) in the genotype-guided group and 32 of 70 (45.7%) in the control group, with no statistically significant between-group difference (χ^2^ = 0.24; *p* = 0.628) (Table 6).

Clinically minor forms of bleeding predominated: bruising was recorded in 47 patients (24 and 23 per group, respectively) and epistaxis in 17 patients (seven and 10, respectively). All remaining bleeding types (gingival, peptic ulcer exacerbation, gastrointestinal, hemorrhoidal, vaginal bleeding, blood in stool, and macrohematuria) occurred only sporadically (1–4 cases over the entire follow-up period) and do not support conclusions about between-group differences for individual categories.

The dynamics of hemorrhagic complications by BARC grade at the three follow-up visits are shown in Table 7. At the 1-month visit, bleeding of any grade was recorded in 34 of 72 patients (47.2%) in the genotype-guided group and 22 of 70 (31.4%) in the control group, predominantly BARC grade 1; clinically relevant bleeding (BARC ≥ 2) occurred in five and six patients, respectively. At the 3-month visit, bleeding rates decreased in both groups (13 and 20 cases, respectively), with BARC ≥ 2 in one and two patients. By the 12-month visit, bleeding persisted in 15 of 71 and 14 of 69 patients, predominantly BARC grade 1, with BARC ≥ 2 in only one patient (genotype-guided group). No visit showed a statistically significant between-group difference in BARC grade distribution (*p* > 0.05 at all visits).

Because the clinical and economic burden of bleeding is primarily driven by events graded BARC ≥ 2 (requiring medical intervention), the 12-month cumulative incidence of such events was chosen as the primary safety endpoint.

**Figure 3:**
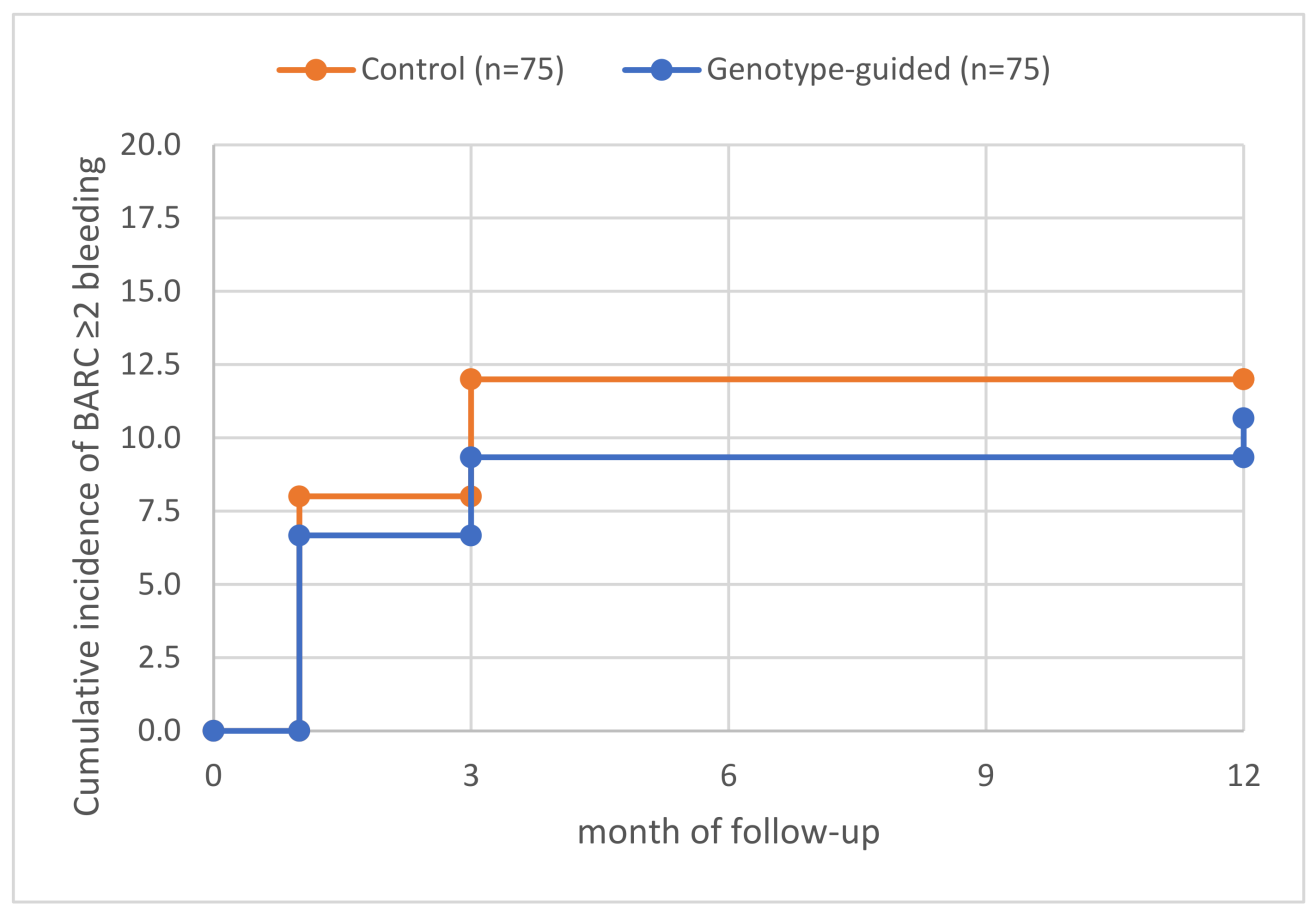
Cumulative incidence of clinically relevant bleeding (BARC ≥ 2).

In the intention-to-treat population (*n* = 150), the 12-month Kaplan–Meier cumulative incidence of BARC ≥ 2 bleeding was 10.7% in the genotype-guided group versus 12.0% in the control group (Figure 3); the difference did not reach statistical significance (log-rank test, *p* = 0.793).

The Cox model HR was 0.88 (95% CI 0.34–2.28; *p* = 0.790). This model was based on 17 events over 1634 patient-months of follow-up; the proportional hazards assumption was not violated (Schoenfeld residual test, *p* = 0.611). A per-protocol sensitivity analysis excluding the eight patients described above (*n* = 142) yielded closely similar estimates (9.7% vs. 11.4%; HR 0.84, 95% CI 0.30–2.32; log-rank *p* = 0.739), indicating that the conclusion for this endpoint is robust to the analytic approach. Although this CI is also wide, the point estimate and its direction were stable across all sensitivity analyses: a supplementary model adjusting for baseline hemoglobin yielded an adjusted HR for the genotype-guided group of 0.84 (95% CI 0.32–2.22); hemoglobin itself was not a significant predictor of bleeding in this model (HR 0.99, 95% CI 0.97–1.02, *p* = 0.66).

### 3.3. DAPT Efficacy

Efficacy of DAPT de-escalation was assessed based on the rate of ischemic events: cardiovascular death and recurrent ischemic episodes (unstable angina, acute coronary syndrome, and ischemia-related rehospitalization) over 12 months of follow-up.

In the per-protocol population, nineteen ischemic events were recorded during follow-up: eight in the genotype-guided group and 11 in the control group (Table 8). In the intention-to-treat population (*n* = 150), 21 ischemic events were recorded: 10 in the genotype-guided group and 11 in the control group.

One death in the control group, confirmed as recurrent myocardial infarction by autopsy, was classified as cardiovascular death. In one further case, in the genotype-guided group, a recurrent myocardial infarction with thrombosis of a previously placed stent was recorded in the first month after enrollment.

The largest share of events was episodes of unstable angina/ACS confirmed by angiography, with a new lesion requiring repeat stenting (three and four cases per group, respectively) or with patent prior stents requiring no new intervention (one and three cases). No statistically significant between-group difference was found in the structure of ischemic events (Fisher’s exact test, *p* = 0.821). Hard events (death, MI, and stent thrombosis) accounted for one event per group; angiographically confirmed ACS/unstable angina accounted for four vs. seven events; unstable angina diagnosed without angiography accounted for three vs. three events (Table 8). Given the small number of hard events, they could not be analyzed separately as a time-to-event endpoint.

**Figure 4:**
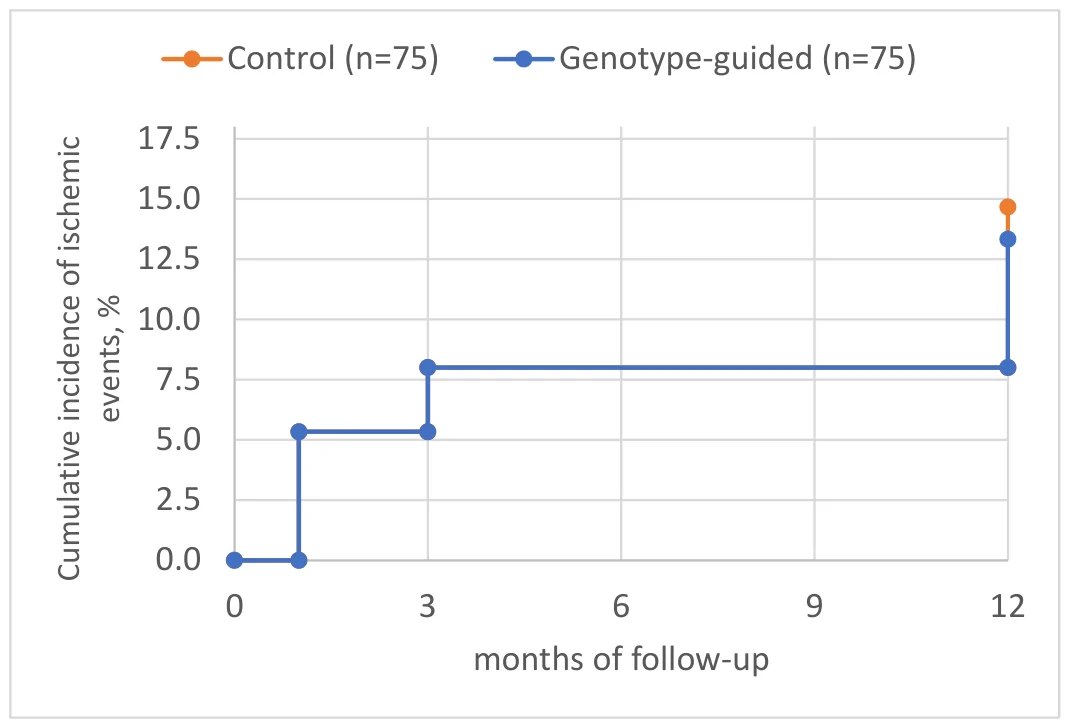
Cumulative incidence of ischemic events over 12 months.

In the intention-to-treat population (*n* = 150), the 12-month Kaplan–Meier cumulative incidence of the ischemic composite was 13.3% in the genotype-guided group versus 14.7% in the control group (Figure 4); the difference did not reach statistical significance (log-rank test, *p* = 0.822).

The Cox model HR was 0.91 (95% CI 0.38–2.13; *p* = 0.822). This model was based on 21 events over 1676 patient-months of follow-up; the proportional hazards assumption was not violated (Schoenfeld residual test, *p* = 0.995). A per-protocol sensitivity analysis (*n* = 142) yielded a numerically larger effect estimate (11.1% vs. 15.7%; HR 0.69, 95% CI 0.28–1.72; log-rank *p* = 0.429); the attenuation in the ITT analysis reflects events among the eight patients who required non-protocol anticoagulant therapy. Both CI values are compatible with a clinically meaningful reduction or increase in ischemic risk; adjustment for baseline imbalance in prior MI reverses the direction of this estimate: a supplementary model yielded HR 1.12 (95% CI 0.46–2.69) for the genotype-guided group, with prior MI itself a significant predictor of ischemic events (HR 3.50, 95% CI 1.42–8.64, *p* = 0.007); adjustment for ACS phenotype (STEMI vs. NSTEMI) yielded HR 0.81 (95% CI 0.34–1.92).

The rates of both planned (18 vs. 16 in the control group, *p* = 0.918) and unplanned (15 vs. 14, *p* = 1.000) hospitalizations did not differ significantly between groups, including the structure of causes of unplanned hospitalizations (Table 9).

Because planned and unplanned hospitalization rates were closely balanced between groups (Table 9), omitted event-related cost categories are unlikely to materially alter the drug cost difference reported below, which was driven by the price differential between ticagrelor and clopidogrel (see Section 3.4).

To assess whether the treatment each patient actually received matched their own genotype recommendation—irrespective of randomization group—all patients were classified into three categories:

- Concordant treatment matched genotype;
- Discordant-conservative patients continued ticagrelor despite a genotype permitting de-escalation to clopidogrel;
- Discordant-risky patients were de-escalated despite carrying loss-of-function CYP2C19 alleles (Table 10).

No statistically significant differences were found in this descriptive, non-randomized comparison between clinical outcomes and concordance categories (Table 11) for either BARC ≥ 2 (12.5% in the concordant group vs. 6.0% in the discordant-conservative group, *p* = 0.356) or ischemic events (13.6% vs. 14.0%, *p* = 1.000).

The absence of statistically significant between-group differences in bleeding rates, recurrent ischemic events, and hospitalization burden is reported above; given the exploratory, underpowered nature of these comparisons (Section 2.4), a cost–consequence analysis was performed to report costs alongside these clinical findings rather than to assume their equivalence.

### 3.4. Cost–Consequence Analysis

Drug and PGx testing costs for the genotype-guided and control groups were calculated and compared as part of the cost–consequence analysis. The input parameters used for the cost calculation (dosing regimens, drug prices, and PGx testing price) are shown in Table 12.

Total drug and PGx testing costs were RUB 1,192,712.45 in the genotype-guided group (RUB 16,565.45 per patient) versus RUB 2,229,052.65 in the control group (RUB 31,843.61 per patient). The saving with the genotype-guided strategy was RUB 1,036,340.21 for the cohort, or RUB 15,278.16 per patient (Table 13).

**Figure 5:**
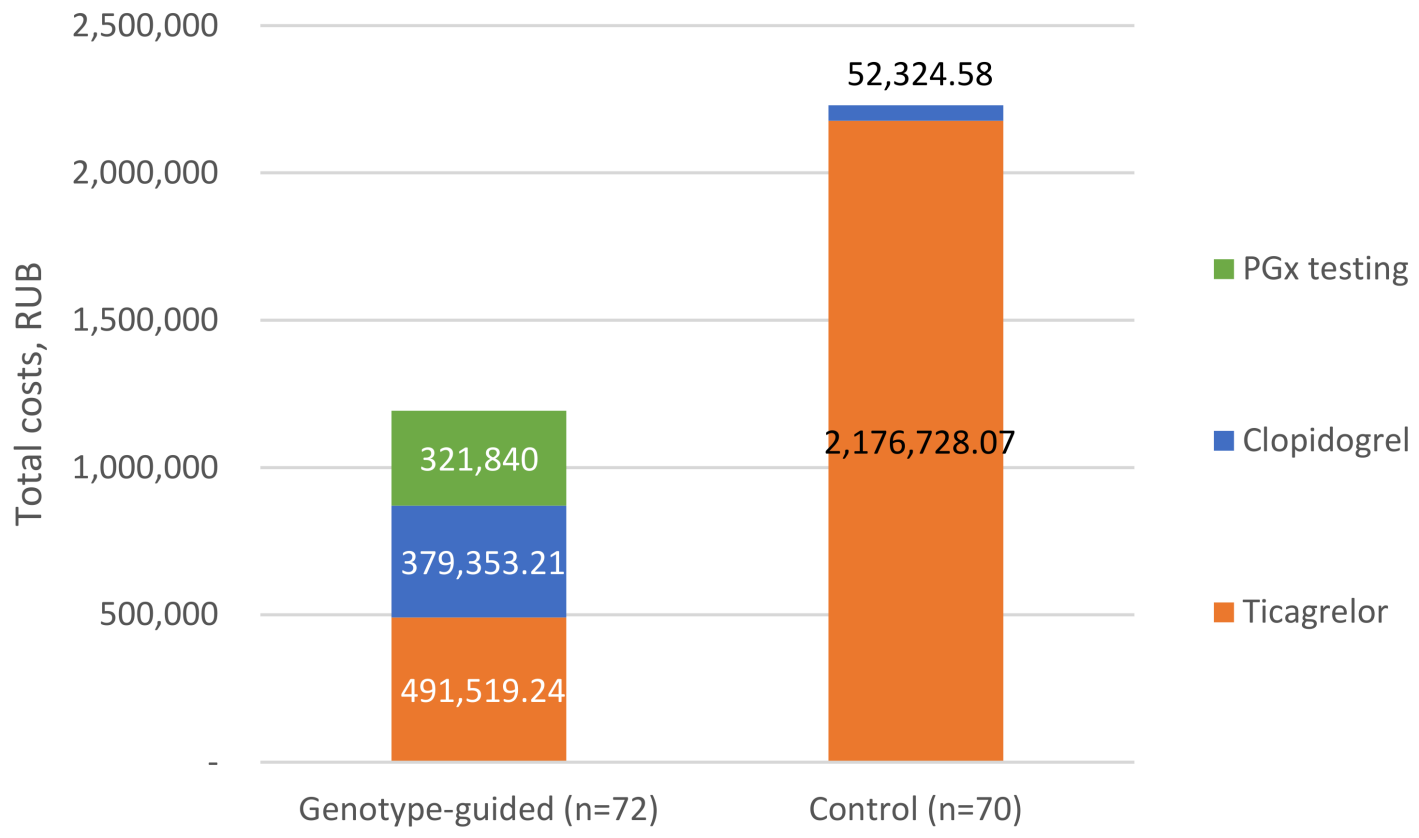
Structure of drug and testing costs by group.

The main driver of the cost difference was drug therapy cost: in the control group, most patients (62 of 70) continued on the more expensive ticagrelor, whereas 58 of 72 patients in the genotype-guided group were switched to clopidogrel (Figure 5).

To assess the robustness of the base case result to uncertainty in key model parameters, a one-way sensitivity analysis was performed: the prices of ticagrelor, clopidogrel, and PGx testing were varied in turn across their observed minimum-to-maximum range (Table 12), with the other two parameters held at their base (median) values. The results are shown in Table 14.

Across all seven scenarios, the cost saving from the genotype-guided strategy remained positive, indicating robustness of the base case result to uncertainty in the price parameters used. The saving ranged from RUB 641,636.21 (at maximum testing price) to RUB 1,713,904.50 (at maximum ticagrelor price) per cohort (Table 14).

**Figure 6:**
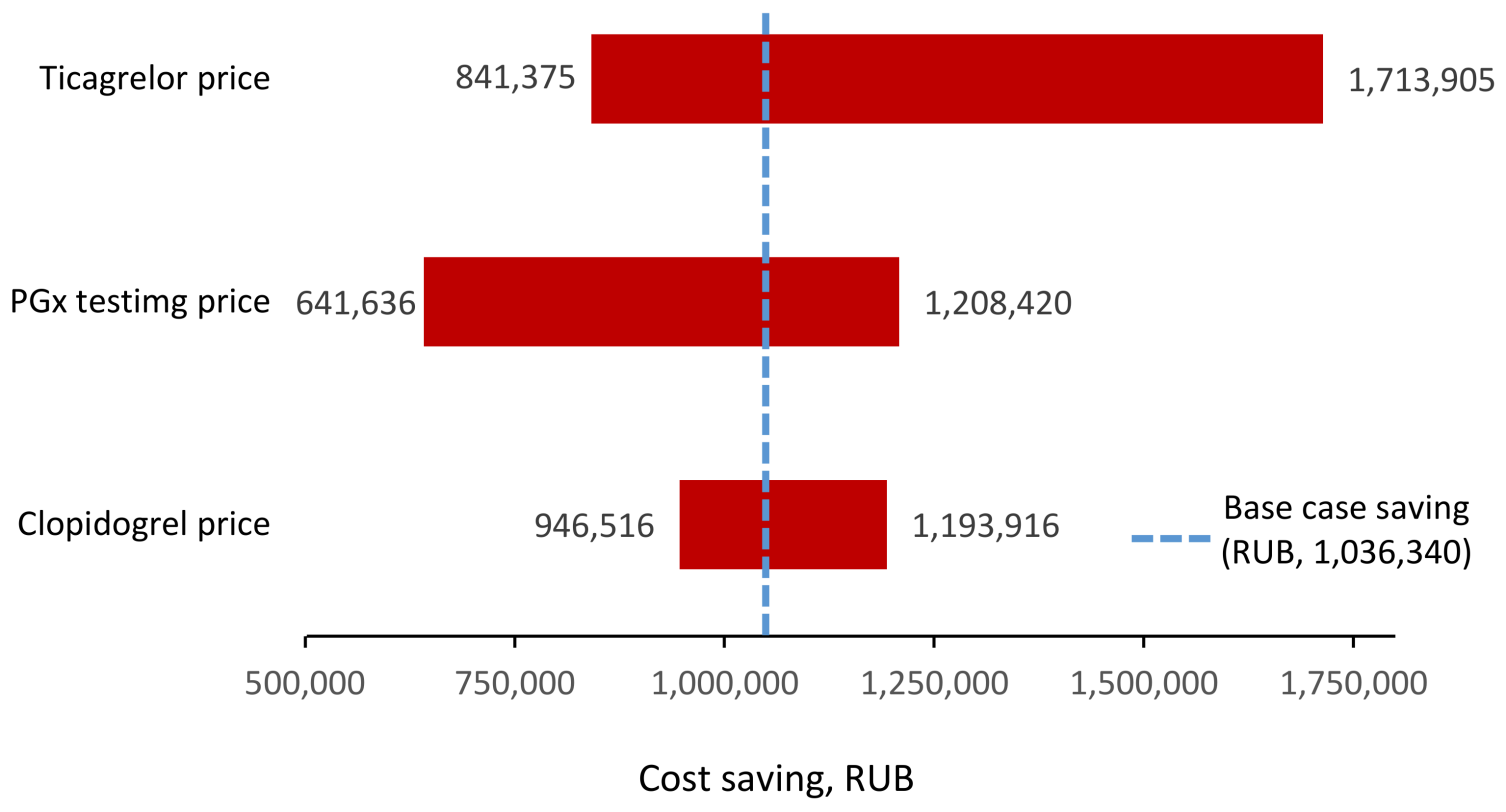
Range of cost savings in the one-way sensitivity analysis.

Ticagrelor price had the greatest influence on the result, reflected in the widest range of variation in savings among the three parameters examined (range width: RUB 872,529), explained by the large proportion of control group patients (62/70) remaining on this drug. PGx testing price had the second-largest influence (range width: RUB 566,784), reflecting the relatively wide variability in prices among laboratories in the Moscow region (RUB 2080–9952). Clopidogrel price had the smallest influence on the result (range width: RUB 247,400) (Figure 6).

**Figure 7:**
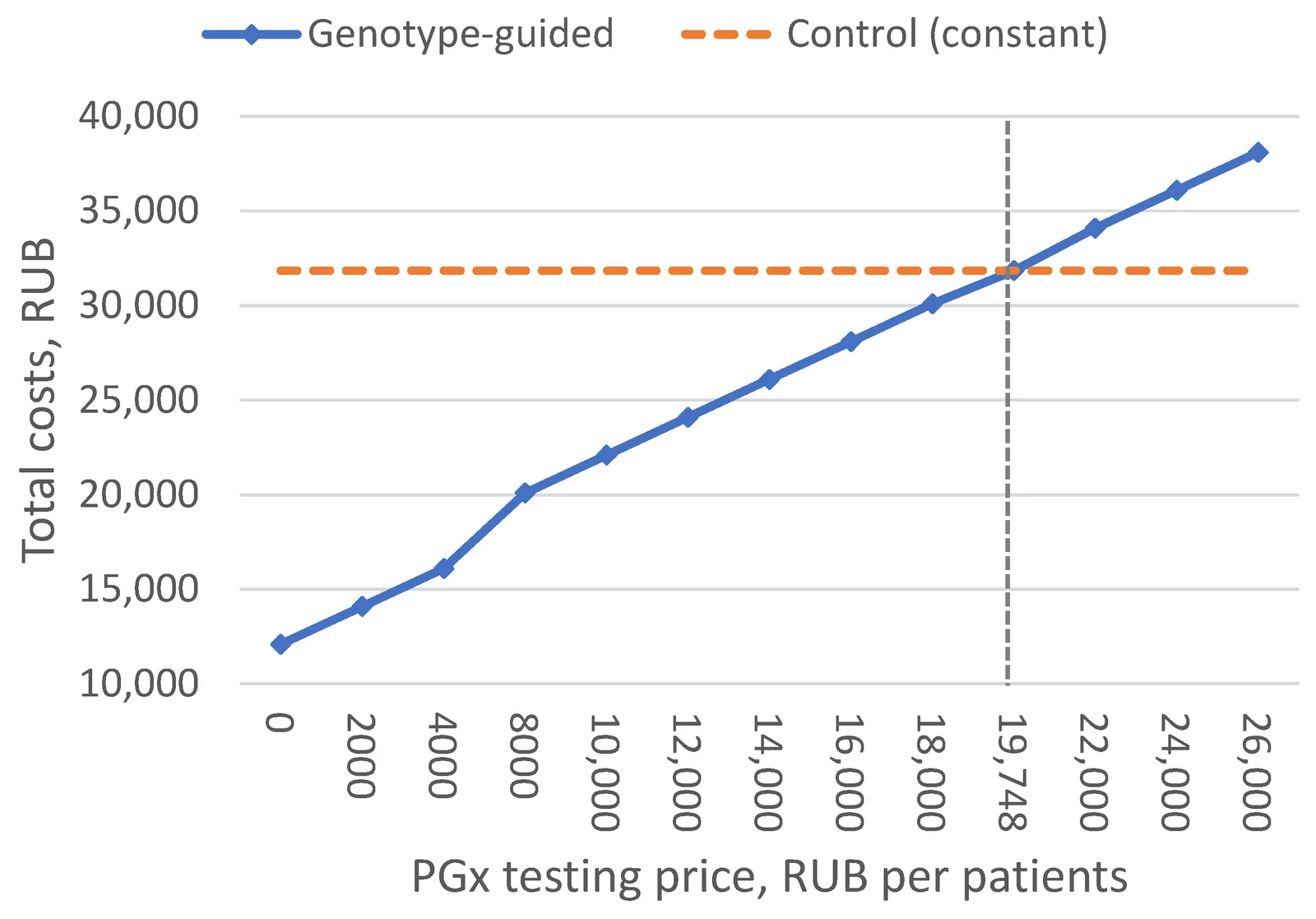
Break-even point for PGx testing price.

A drug-and-testing-cost break-even price—the testing price at which the drug-and-testing-cost saving from the genotype-guided strategy becomes zero—was additionally determined. This threshold was RUB 19,748.16 per patient (Figure 7).

## 4. Discussion

In the intention-to-treat population, genotype-guided de-escalation of DAPT based on CYP2C19 testing was associated with numerically lower, though not statistically significant, rates of both hemorrhagic and ischemic events compared with standard clinical practice: the 12-month cumulative incidence of clinically relevant bleeding (BARC ≥ 2) was 10.7% versus 12.0% (log-rank *p* = 0.793; HR 0.88, 95% CI 0.34–2.28), and the cumulative incidence of ischemic events was 13.3% versus 14.7% (*p* = 0.822; HR 0.91, 95% CI 0.38–2.13). A per-protocol sensitivity analysis excluding eight patients who required non-protocol anticoagulant therapy showed a closely similar estimate for bleeding (HR 0.84) and a numerically larger, though still non-significant, effect for the ischemic composite (HR 0.69) (see the paragraph discussing limitations below). Hospitalization burden also did not differ significantly between groups (*p* = 1.000).

Our finding of a trend toward reduced BARC ≥ 2 incidence in the genotype-guided group (9.7% vs. 11.4%, HR 0.84, 95% CI 0.30–2.32) parallels the reduction in bleeding reported in POPular Genetics, where CYP2C19 genotype-guided de-escalation was associated with a statistically significant reduction in bleeding (9.8% vs. 12.5%, HR 0.78; 95% CI 0.61–0.98; *p* = 0.04), with comparable rates of ischemic events [19]. A bleeding reduction in a similar direction, though achieved through different, non-genotype-guided mechanisms, was also reported in TALOS-AMI, where de-escalation from ticagrelor to clopidogrel was performed without genetic or platelet function guidance (BARC 2, 3, or 5 composite 3.0% vs. 5.6%, HR 0.52, 95% CI 0.35–0.77, *p* = 0.0012) [27]; in TROPICAL-ACS, where de-escalation from prasugrel to clopidogrel was guided by platelet function testing (composite primary endpoint 7% vs. 9%) [28]; and in HOST-REDUCE-POLYTECH-ACS, which evaluated a prasugrel dose reduction rather than a switch between agents (primary endpoint was 7.2% vs. 10.1%, RR 0.70, 95% CI 0.52–0.92; bleeding risk RR 0.48, 95% CI 0.32–0.73, *p* = 0.0007, with no corresponding increase in ischemic risk, HR 0.76, 95% CI 0.40–1.45, *p* = 0.40) [29]. Because a reduction in bleeding was observed regardless of whether de-escalation was guided by genotype, platelet function, dose, or no biomarker at all, this pattern likely reflects a general consequence of reduced antiplatelet intensity rather than evidence specific to genotype-guided selection; the substantially larger sample sizes in these trials (2338–2610 patients, versus 150 in the present study) most plausibly account for their statistical significance relative to our own finding (Table 15). The direction of effect for the ischemic composite (11.1% vs. 15.7%, RR 0.69, 95% CI 0.28–1.72) is consistent with TAILOR-PCI, which directly compared CYP2C19 genotype-guided DAPT with conventional therapy (clopidogrel in all patients, without a ticagrelor continuation arm; Table 15). Its primary ischemic endpoint—comprising cardiovascular death, MI, stroke, stent thrombosis, and severe recurrent ischemia—also did not reach statistical significance at 12 months (4.0% vs. 5.9%, HR 0.66, 95% CI 0.43–1.02, *p* = 0.06) [20]. A similar non-significant trend was reported for the ischemic sub-composite of cardiovascular death, MI, and stroke in TALOS-AMI (2.1% vs. 3.1%, HR 0.69, 95% CI 0.42–1.14, *p* = 0.15) [27], although that trial tested unguided rather than genotype-guided de-escalation. This pattern—a non-significant ischemic endpoint alongside a favorable numerical trend—recurs even in trials with substantially larger sample sizes, and likely reflects a general limitation of statistical power for relatively rare ischemic events in this field rather than a specific replication of a genotyping effect. Given the width of these CIs, non-significance should not be interpreted as evidence of no effect. Consistent with recommendations for exploratory, pilot-stage trials [30], we treat the ischemic findings of the present study as hypothesis-generating rather than confirmatory.

Because clinical equivalence between strategies could not be formally established in this pilot trial, we report cost findings descriptively rather than inferring economic superiority. The cost–consequence analysis shows that the genotype-guided strategy was associated with substantial drug cost savings, driven by more frequent de-escalation to clopidogrel, which exceeded the cost of PGx testing. Whether this cost advantage translates into a favorable overall value proposition depends on the still-uncertain comparative clinical outcomes discussed above. Because the trial randomized a decision strategy rather than a drug, this cost difference is substantially attributable to the large gap in treatment exposure the strategy produced (Table 5) rather than to an independent effect of genotyping. This result is driven primarily by the higher price of ticagrelor, in the absence of any offsetting difference in the rate of hemorrhagic or ischemic complications requiring additional medical intervention.

The robustness of this estimate to price uncertainty is supported by the one-way sensitivity analysis: the cost saving remained positive across the full range of observed drug prices (RUB 641,636.21 to 1,713,904.50 per cohort), supporting the reliability of the conclusion regarding the cost advantage of the genotype-guided approach.

Further support comes from the calculated drug-and-testing-cost break-even price for PGx testing, RUB 19,748.16 per patient, which is almost 4.4 times the actual testing price (RUB 4470). This substantial margin indicates that the economic advantage of the genotype-guided strategy would persist even with a marked increase in PGx testing price. Conversely, given the observed price variability, this threshold could serve as a practical benchmark for healthcare organizers selecting a genetic testing provider.

This finding is consistent with international cost-effectiveness studies showing that CYP2C19 genotype-guided personalization of antiplatelet therapy is cost-effective primarily through drug price differences rather than the prevention of clinical events: in the systematic review by AlMukdad et al. (2020), which analyzed 13 studies, a genotype-guided strategy was found to be cost-effective in six studies and dominant in five, compared with universal ticagrelor or prasugrel use [31]. The most closely comparable analysis in design is that of van den Broek et al. (2024), based on the FORCE-ACS registry: a genotype-guided de-escalation strategy was cost-saving relative to continued standard DAPT in 96% of simulations in a probabilistic sensitivity analysis, with a concurrent gain in quality-adjusted life years (QALYs) in 87% of simulations [32]. Similar findings regarding the cost-effectiveness of a genotype-guided approach, driven by reduced drug expenditure at comparable clinical efficacy, have been reported in other national healthcare systems: the Netherlands (economic sub-analysis of POPular Genetics) [33], Spain [34], Serbia [35], and Hong Kong [36]. No dedicated cost-effectiveness studies of DAPT de-escalation in ACS patients have previously been conducted in the Russian Federation, precluding direct comparison with Russian data and underscoring the novelty of the present study for the Russian healthcare system.

Taken together, these findings indicate that genotype-guided DAPT de-escalation based on CYP2C19 testing is associated with substantially lower drug and testing costs than standard practice, which is driven by drug price differences rather than by a demonstrated reduction in clinical event rates. The comparability of these factors remains unresolved in this pilot trial. Confirming or refuting comparable clinical safety and efficacy, and establishing a full cost-effectiveness profile using QALYs, will require further studies with larger sample sizes and a multicenter design, providing adequate power to assess differences in rare clinical events. The contribution of additional pharmacogenetic markers potentially associated with the pharmacodynamic and pharmacokinetic parameters of DAPT drugs (e.g., ABCB1, CYP3A4/5, CYP2B6, and others) warrants separate study.

This study has several limitations that should be considered when interpreting the findings. First, the study was exploratory in nature: the sample size was determined by the practical constraints of recruitment at a single center over 1 year, without an a priori power calculation for specific clinical endpoints. A post hoc assessment shows limited power for relatively rare binary clinical events (bleeding, ischemic events). With 17–21 events per Cox model, conventional asymptotic inference may be imprecise; small-sample corrections (e.g., Firth-penalized regression) were not applied, and the absence of separation does not by itself establish reliability. This limitation is shared by considerably larger trials in this field: in TAILOR-PCI, with a sample size of 5302 patients, the primary ischemic endpoint did not reach statistical significance (*p* = 0.06) [20], pointing to a broader methodological challenge in demonstrating hard clinical outcomes for genotype-guided interventions in this field. The single-center design further limits generalizability to patient populations with different clinical and demographic characteristics and care settings. Second, the effect estimate for the ischemic composite was less robust to the analytic approach than for the primary safety endpoint: the intention-to-treat analysis (primary; HR 0.91, 95% CI 0.38–2.13) showed a more attenuated effect than the per-protocol sensitivity analysis (HR 0.69, 95% CI 0.28–1.72), driven by events among the eight patients who required non-protocol anticoagulant therapy, including two borderline ischemic events. In contrast, the estimate for the primary safety endpoint (BARC ≥ 2) was materially unchanged between the ITT and per-protocol analyses (HR 0.88 and 0.84, respectively), supporting the robustness of this conclusion. A post hoc sensitivity analysis adjusting for baseline hemoglobin left the bleeding estimate materially unchanged (HR 0.84), whereas adjusting for the observed imbalance in prior MI history attenuated and reversed the direction of the ischemic estimate (HR from 0.91 to 1.12), indicating that the ischemic endpoint’s apparent direction of effect is not robust to plausible baseline confounding. Third, the study lacks objective data on actual adherence to prescribed antiplatelet therapy (e.g., pharmacy dispensing records or pill counts) between scheduled visits; adherence was inferred from patient self-report during in-person and telephone follow-up. Reasons for treatment discontinuation or switching were documented only for cases involving a new clinical indication (Section 3.1) and not systematically captured otherwise; persistence with the assigned P2Y12 inhibitor by visit is reported in Table 5. Fourth, because clinical equivalence between strategies could not be established in this pilot trial, we deliberately used a cost–consequence framework. The cost of PGx testing was based on retail prices for individual patients published on the websites of Moscow-region laboratories as of August 2026, and may not reflect the actual cost under a contract between a healthcare institution and a laboratory, which is typically lower than retail pricing when arranged through a network provider; in that case, actual savings may exceed the calculated estimate. The analysis reflects a drug-and-testing-cost perspective. Costs related to hospitalization, revascularization, bleeding management, monitoring, or test implementation were not included. Finally, the contribution of drug–drug interactions potentially affecting CYP2C19, CYP3A4, and P-glycoprotein activity was not assessed in this work and is the subject of a separate publication.

## 5. Conclusions

In this study, no statistically significant differences were found in the rates of hemorrhagic and ischemic complications between genotype-guided CYP2C19-based DAPT de-escalation and standard clinical practice in patients after ACS and PCI. The bleeding result was stable across the intention-to-treat and per-protocol analyses and after adjustment for baseline hemoglobin; however, the ischemic result was not stable; it reversed after adjustment for the imbalance in prior MI between groups, and should thus be treated as hypothesis-generating.

The cost–consequence analysis showed that the genotype-guided strategy is associated with meaningful drug cost savings that are robust to variation in key price parameters, independent of the still-uncertain comparative clinical outcomes reported above. Together, these findings support genotype-guided DAPT de-escalation based on CYP2C19 testing as a cost-saving strategy. However, wider adoption in routine clinical practice will require further evaluation in prospective, larger, multicenter studies incorporating quality-of-life outcomes.

## Figures and Tables

**Table 1:** Clinical and demographic characteristics of the study cohort and comparison by groups.

Parameter	Cohort (*n* = 150)	Genotype-Guided (*n* = 75)	Control (*n* = 75)	*p*-Value
Demographics and anthropometrics				
Male	100 (66.7%)	52 (69.3%)	48 (64.0%)	0.489
Female	50 (33.3%)	23 (30.7%)	27 (36.0%)	
Age, years	64.00 [54.25;69.00]	65.00 [59.00;70.00]	63.00 [52.50;68.00]	0.206
Smoking	79 (52.7%)	38 (50.7%)	41 (54.7%)	0.744
BMI, kg/m^2^	27.76 [25.22;31.22]	28.08 [25.48;31.18]	27.68 [25.23;32.28]	0.889
Length of hospital stay, days	6.00 [5.00;6.00]	6.00 [5.00;6.00]	6.00 [5.00;6.00]	0.520
Comorbidities				
Prior MI	26 (17.3%)	8 (10.7%)	18 (24.0%)	0.052
Prior stenting	22 (14.7%)	8 (10.7%)	14 (18.7%)	0.249
Prior CABG	3 (2.0%)	2 (2.7%)	1 (1.3%)	1.000
Prior stroke	6 (4.0%)	3 (4.0%)	3 (4.0%)	1.000
Hypertension	127 (84.7%)	64 (85.3%)	63 (84.0%)	1.000
Diabetes mellitus	35 (23.3%)	16 (21.3%)	19 (25.3%)	0.699
Prior therapy				
β-blockers	44 (29.3%)	21 (28.0%)	23 (30.7%)	0.858
ACE inhibitors	36 (24.0%)	20 (26.7%)	16 (21.3%)	0.566
ARBs	31 (20.7%)	14 (18.7%)	17 (22.7%)	0.687
Statins	30 (20.0%)	12 (16.0%)	18 (24.0%)	0.307
Aspirin	39 (26.0%)	14 (18.7%)	25 (33.3%)	0.063
Clopidogrel	3 (2.0%)	1 (1.3%)	2 (2.7%)	1.000
Ticagrelor	9 (6.0%)	3 (4.0%)	6 (8.0%)	0.494
Severity and diagnosis at admission				
Killip I/II/III/IV, *n*	127/12/5/6	63/7/3/2	64/5/2/4	0.801
STEMI	63 (42.0%)	26 (34.7%)	37 (49.3%)	0.069
NSTEMI	87 (58%)	49 (65.3%)	38 (50.7%)	
Angiographic characteristics				
Number of diseased vessels (1/2/3), *n*	35/45/70	20/25/30	15/20/40	0.259
Left main stenting	3 (2.0%)	2 (2.7%)	1 (1.3%)	1.000
LAD stenting	71 (47.3%)	38 (50.7%)	33 (44.0%)	0.513
LCx stenting	31 (20.7%)	14 (18.7%)	17 (22.7%)	0.687
OM stenting	1 (0.7%)	0 (0.0%)	1 (1.3%)	1.000
RCA stenting	49 (32.7%)	22 (29.3%)	27 (36.0%)	0.486
Laboratory parameters				
Hemoglobin, g/L	144.33 ± 17.53	140.83 ± 18.14	147.83 ± 16.28	0.014 *
Erythrocytes, ×10^12^/L	4.76 ± 0.62	4.64 ± 0.57	4.88 ± 0.64	0.015 *
Leukocytes, ×10^9^/L	10.37 [8.71;13.00]	10.41 [8.60;12.74]	10.32 [8.73;13.56]	0.430
Platelets, ×10^9^/L	244.50 [200.00;284.50]	238.00 [187.00;276.50]	253.00 [218.50;286.00]	0.138
Total cholesterol, mmol/L	5.60 [4.80;6.60]	5.50 [4.60;6.20]	5.60 [5.00;6.65]	0.348
LDL, mmol/L (*n* = 147)	3.64 ± 1.39	3.59 ± 1.51	3.68 ± 1.29	0.699
HDL, mmol/L (*n* = 146)	1.17 [0.96;1.45]	1.24 [0.95;1.46]	1.12 [0.99;1.37]	0.410
Triglycerides, mmol/L	1.56 [1.12;2.34]	1.49 [1.07;2.28]	1.63 [1.19;2.33]	0.125
Creatinine, μmol/L	89.00 [78.25;103.00]	90.00 [80.50;100.50]	88.00 [78.00;105.50]	0.909
eGFR (CKD-EPI), mL/min/1.73 m^2^	84.50 [69.00;95.00]	83.00 [70.00;94.00]	87.00 [69.00;97.00]	0.835
Glucose, mmol/L	7.10 [6.00;9.00]	7.10 [5.90;9.65]	7.10 [6.00;8.70]	0.937
LVEF, %	50.00 [44.00;55.00]	52.00 [44.50;56.00]	50.00 [44.00;55.00]	0.228
SBP, mmHg	130.00 [120.00;145.00]	130.00 [120.00;140.00]	130.00 [120.00;150.00]	0.580
DBP, mmHg	80.00 [78.50;90.00]	80.00 [70.00;90.00]	80.00 [80.00;90.00]	0.578
Heart rate, bpm	68.50 [65.00;79.00]	68.00 [65.00;78.00]	72.00 [67.00;82.50]	0.048 *

Notes: LAD—left anterior descending artery; LCx—left circumflex artery; OM—obtuse marginal branch; RCA—right coronary artery; LDL—low-density lipoprotein; HDL—high-density lipoprotein; LVEF—left ventricular ejection fraction; SBP—systolic blood pressure; and DBP—diastolic blood pressure. * *p* < 0.05, statistically significant difference.

**Table 2:** Frequency of CYP2C19 allelic variants in the study sample.

Marker	Genotype	Observed	Expected	%	MAF, %	χ^2^	*p*-Value
CYP2C19*2 (rs4244285)	GG	121	119.7	80.7	10.7	1.228	0.268
	GA	26	28.6	17.3			
	AA	3	1.7	2.0			
CYP2C19*3 (rs4986893)	GG	149	149.0	99.3	0.3	NA	NA
	GA	1	1.0	0.7			
	AA	0	0	0.0			
CYP2C19*17 (rs12248560)	CC	83	82.1	55.3	26.0	0.133	0.715
	CT	56	57.7	37.3			
	TT	11	10.1	7.4			

Notes: MAF—minor allele frequency.

**Table 3:** Distribution of CYP2C19 phenotypes by group.

Genotypes	Phenotype	Genotype-Guided, *n* (%)	Control, *n* (%)	*p*-Value
*17/*17	UMs	8 (10.67)	3 (4.0)	0.489
*1/*17	RMs	21 (28.0)	27 (36.0)	
*1/*1	NMs	32 (42.67)	29 (38.67)	
*1/*2, *1/*3, *2/*17	IMs	13 (17.33)	14 (18.67)	
*2/*2	PMs	1 (1.33)	2 (2.67)	

Notes: UMs—ultrarapid metabolizers; RMs—rapid metabolizers; NMs—normal metabolizers; IMs—intermediate metabolizers; and PMs—poor metabolizers.

**Table 4:** Concordance between the actual DAPT de-escalation decision and CYP2C19 genotyping results, by study group (per-protocol population).

Parameter	Genotype-Guided (*n* = 72)	Control (*n* = 70)
De-escalation indicated by CYP2C19 (NM/RM/UM)	58 (80.6%)	54 (77.1%)
Actually de-escalated (Visit 2, 1 month)	58 (80.6%)	8 (11.4%)
Analysis against genotyping results		
Concordant with genotype	70 (97.2%)	18 (25.7%)
Not de-escalated despite CYP2C19 indication	1 (1.4%) *	49 (70.0%)
De-escalated despite CYP2C19 indication to continue ticagrelor (IM)	1 (1.4%) *	3 (4.3%)

Note: *—study protocol deviation.

**Table 5:** Actual P2Y12 inhibitor therapy received at each follow-up visit (per-protocol population).

Visit	Genotype-Guided (*n* = 72) Clopidogrel	Genotype-Guided (*n* = 72) Ticagrelor	Control (*n* = 70) Clopidogrel	Control (*n* = 70) Ticagrelor
Visit 2 (1 month)	0 (0.0%)	72 (100.0%)	0 (0.0%)	70 (100.0%)
Visit 3 (3 months)	57 (79.2%)	15 (20.8%)	9 (12.9%)	61 (87.1%)
Visit 4 (12 months)	51 (70.8%)	14 (19.4%)	8 (11.4%)	55 (78.6%)

**Table 6:** Structure of bleeding events over 12 months of follow-up (per-protocol population).

Bleeding Type	N	Genotype-Guided (*n* = 72)	Control (*n* = 70)
Bruising	47	24 (33.3%)	23 (32.9%)
Epistaxis	17	7 (9.7%)	10 (14.3%)
Gingival bleeding	4	2 (2.8%)	2 (2.9%)
Peptic ulcer exacerbation	2	0 (0.0%)	2 (2.9%)
Severe epistaxis	1	1 (1.4%)	0 (0.0%)
GI bleeding	1	1 (1.4%)	0 (0.0%)
Hemorrhoidal bleeding	1	1 (1.4%)	0 (0.0%)
Vaginal bleeding	1	0 (0.0%)	1 (1.4%)
Blood in stool	1	1 (1.4%)	0 (0.0%)
Macrohematuria	1	0 (0.0%)	1 (1.4%)
Any bleeding, total	61	29 (40.3%)	32 (45.7%)

**Table 7:** Distribution of bleeding events by BARC grade, per visit (per-protocol population).

Visit	BARC	Genotype-Guided (*n* = 72)	Control (*n* = 70)	*p*-Value for BARC ≥ 2 Comparison
Visit 2 (1 month)	0	38	48	0.095
	1	29	16	
	2	4	6	
	3a	1	0	
Visit 3 (3 months)	0	59	50	0.137
	1	12	18	
	2	1	2	
Visit 4 (12 months) *	0	56	55	0.873
	1	14	14	
	3a	1	0	

Note: *—numbers of patients at the 12-month visit (71 and 69) reflect one death per group before that visit.

**Table 8:** Structure of ischemic events by group (per-protocol population).

Event Category	Genotype-Guided (*n* = 72)	Control (*n* = 70)
Death (CV death, recurrent MI confirmed at autopsy)	0	1
Recurrent MI with stent thrombosis	1	0
ACS/unstable angina, angiography confirmed new lesion—new stenting/re-stenting required	3	4
ACS/unstable angina, angiography performed, stents patent—no new intervention required	1	3
Unstable angina, no repeat angiography performed (clinical diagnosis)	3	3
Total	8	11

Notes: CV—cardiovascular; MI—myocardial infarction; and ACS—acute coronary syndrome.

**Table 9:** Structure of hospitalizations over 12 months of follow-up.

Parameter	Genotype-Guided (*n* = 72)	Control (*n* = 70)	*p*-Value
Planned hospitalization	18 (25.0%)	16 (22.9%)	0.918
Any unplanned hospitalization	15 (20.8%)	14 (20.0%)	1.000
—Ischemic cause	8 (11.1%)	10 (14.3%)	
—Hemorrhagic cause	2 (2.8%)	0 (0.0%)	
—Other cause	5 (6.9%)	5 (7.1%)	

**Table 10:** Distribution of concordance categories.

Category	N	Genotype-Guided (*n* = 72)	Control (*n* = 70)
Concordant	88	70	18
Discordant-conservative	50	1	49
Discordant-risky	4	1	3

**Table 11:** Clinical outcomes by genotype treatment concordance category.

Category	N	BARC ≥ 2, *n* (%)	Ischemic Events, *n* (%)
Concordant	88	11 (12.5%)	12 (13.6%)
Discordant-conservative	50	3 (6.0%)	7 (14.0%)
Discordant-risky	4	1 (25.0%)	0 (0.0%)

**Table 12:** Input parameters for the cost analysis.

Parameter	Ticagrelor	Clopidogrel
Daily dose, mg	180	75
Days per month, mean	30.5	30.5
Price per mg, RUB (median [min; max]) *	0.581 [0.514; 0.815]	0.260 [0.135; 0.331]
Monthly drug therapy cost, RUB	3191.68	594.60
PGx testing cost per patient, RUB (median [min; max]) **	4470 [2080; 9952]	

Notes: *—Prices from the GRPOTs register; **—prices for CYP2C19 panel testing (*2, *3, and *17 alleles). Prices were sourced from laboratories in the Moscow region in August 2026.

**Table 13:** Structure and total costs of drug therapy and PGx testing by group (11 months).

Parameter	Genotype-Guided (*n* = 72)	Control (*n* = 70)
Patients on ticagrelor, *n*	14	62
Patients on clopidogrel, *n*	58	8
Ticagrelor cost, RUB	491,519.24	2,176,728.07
Clopidogrel cost, RUB	379,353.21	52,324.58
Total drug therapy cost, RUB	870,872.45	2,229,052.65
PGx testing cost, RUB	321,840	—
Total cost, RUB	1,192,712.45	2,229,052.65
Cost per patient, RUB	16,565.45	31,843.61

**Table 14:** One-way sensitivity analysis results.

#	Scenario	Ticagrelor *	Clopidogrel *	Testing	Genotype-Guided (*n* = 72)	Control (*n* = 70)	Difference
1	Base case	0.581	0.260	4470	1,192,712.45	2,229,052.65	1,036,340.21
2	Max. ticagrelor price	0.815	0.260	4470	1,390,335.37	3,104,239.87	1,713,904.50
3	Min. ticagrelor price	0.514	0.260	4470	1,135,847.71	1,977,223.12	841,375.41
4	Max. clopidogrel price	0.581	0.331	4470	1,296,908.44	2,243,424.52	946,516.07
5	Min. clopidogrel price	0.581	0.135	4470	1,009,924.33	2,203,840.50	1,193,916.17
6	Max. testing price	0.581	0.260	9952	1,587,416.45	2,229,052.65	641,636.21
7	Min. testing price	0.581	0.260	2080	1,020,632.45	2,229,052.65	1,208,420.21

Note: In each scenario, only one parameter is varied; the other two are held at base case values. *—price per mg of the active substance in RUB from the GRPOTs register.

**Table 15:** Comparison of the present trial with cited de-escalation/genotype-guided RCTs.

Trial	N Randomized	Population	Guidance Tactic	Intervention	Timing	Primary Endpoint	Follow-Up	Key Result
Present study	150	ACS (42% STEMI/58% NSTEMI), post-PCI	CYP2C19 genotype-guided	Ticagrelor->clopidogrel de-escalation vs. continued ticagrelor (physician judgment)	De-escalation at 1-month post-PCI	BARC ≥ 2 bleeding (safety); ischemic composite (efficacy)	12 mo	HR 0.88 (bleeding, ITT); HR 0.91 (ischemia, ITT)
POPular Genetics [19]	2488	100% STEMI, primary PCI	CYP2C19 genotype-guided	Genotype-guided P2Y12 selection (LOF carriers->ticagrelor/prasugrel; non-carriers->clopidogrel) vs. standard ticagrelor/prasugrel	Selection at enrollment (≤48 h post-PCI); not a de-escalation	Co-primary: net clinical events (death/MI/stent thrombosis/stroke/PLATO major bleeding); PLATO major/minor bleeding	12 mo	Bleeding 9.8% vs. 12.5%, HR 0.78 (0.61–0.98); ischemic non-inferior
TAILOR-PCI [20]	5302 (903 vs. 946 LOF carriers analyzed)	82% ACS, 18% stable CAD	CYP2C19 genotype-guided	Genotype-guided selection (LOF carriers->ticagrelor; non-carriers->clopidogrel) vs. clopidogrel for all (no ticagrelor continuation arm)	Selection at enrollment; not a de-escalation	CV death, MI, stroke, stent thrombosis, or severe recurrent ischemia (in LOF carriers)	12 mo	4.0% vs. 5.9%, HR 0.66 (0.43–1.02)
TALOS-AMI [27]	2697	100% AMI (STEMI+NSTEMI), stabilized	Unguided (no genetic/platelet testing)	Ticagrelor->clopidogrel de-escalation (all patients) vs. continued ticagrelor	De-escalation at 1-month post-PCI	CV death, MI, stroke, or BARC 2/3/5 bleeding	1–12 mo post-randomization	4.6% vs. 8.2% (composite); bleeding-driven
TROPICAL-ACS [28]	2610	Biomarker-positive ACS, post-PCI	Platelet function-guided	Prasugrel->clopidogrel de-escalation (if no high platelet reactivity) vs. continued prasugrel	De-escalation at 14 days post-discharge	CV death, MI, stroke, or BARC ≥ 2 bleeding	12 mo	7% vs. 9%
HOST-REDUCE-POLYTECH-ACS [29]	2338	East Asian ACS, post-PCI	Unguided, dose-based (no drug switch)	Prasugrel dose reduction 10->5 mg vs. continued 10 mg	Dose reduction at 1-month post-PCI	Death, MI, stent thrombosis, revascularization, stroke, or BARC ≥ 2 bleeding	12 mo	7.2% vs. 10.1%, HR 0.70 (0.52–0.92)

## Data Availability

The datasets generated and analyzed during this study are not publicly available due to ethical restrictions and patient confidentiality protections under Russian Federation laws on personal data protection (Federal Law No. 152-FZ). However, anonymized data supporting the findings may be made available upon reasonable request from qualified researchers. Requests should include a detailed research proposal and data protection plan.

## Abbreviations

The following abbreviations are used in this manuscript:

ACS	Acute coronary syndrome
BARC	Bleeding Academic Research Consortium
CI	Confidence interval
CCA	Cost–consequence analysis
CPIC	Clinical Pharmacogenetics Implementation Consortium
DAPT	Dual antiplatelet therapy
GRPOTs	State Register of Maximum Manufacturer Selling Prices (Russia)
HR	Hazard ratio
MI	Myocardial infarction
PCI	Percutaneous coronary intervention
PGx	Pharmacogenetic(s)
QALYs	Quality-adjusted life years
